# Harnessing the Endocannabinoid 2-Arachidonoylglycerol to Lower Intraocular Pressure in a Murine Model

**DOI:** 10.1167/iovs.16-19356

**Published:** 2016-06-22

**Authors:** Sally Miller, Emma Leishman, Sherry Shujung Hu, Alhasan Elghouche, Laura Daily, Natalia Murataeva, Heather Bradshaw, Alex Straiker

**Affiliations:** 1 Department of Psychological and Brain Sciences Indiana University, Bloomington, Indiana, United States; 2 Department of Psychology, National Cheng Kung University, Tainan, Taiwan

**Keywords:** intraocular pressure, eye, cannabinoid, endocannabinoid, glaucoma, 2-AG, 2-arachidonoyl glycerol

## Abstract

**Purpose:**

Cannabinoids, such as Δ^9^-THC, act through an endogenous signaling system in the vertebrate eye that reduces IOP via CB_1_ receptors. Endogenous cannabinoid (eCB) ligand, 2-arachidonoyl glycerol (2-AG), likewise activates CB_1_ and is metabolized by monoacylglycerol lipase (MAGL). We investigated ocular 2-AG and its regulation by MAGL and the therapeutic potential of harnessing eCBs to lower IOP.

**Methods:**

We tested the effect of topical application of 2-AG and MAGL blockers in normotensive mice and examined changes in eCB-related lipid species in the eyes and spinal cord of MAGL knockout (MAGL^−/−^) mice using high performance liquid chromatography/tandem mass spectrometry (HPLC/MS/MS). We also examined the protein distribution of MAGL in the mouse anterior chamber.

**Results:**

2-Arachidonoyl glycerol reliably lowered IOP in a CB_1_- and concentration-dependent manner. Monoacylglycerol lipase is expressed prominently in nonpigmented ciliary epithelium. The MAGL blocker KML29, but not JZL184, lowered IOP. The ability of CB_1_ to lower IOP is not desensitized in MAGL^−/−^ mice. Ocular monoacylglycerols, including 2-AG, are elevated in MAGL^−/−^ mice but, in contrast to the spinal cord, arachidonic acid and prostaglandins are not changed.

**Conclusions:**

Our data confirm a central role for MAGL in metabolism of ocular 2-AG and related lipid species, and that endogenous 2-AG can be harnessed to reduce IOP. The MAGL blocker KML29 has promise as a therapeutic agent, while JZL184 may have difficulty crossing the cornea. These data, combined with the relative specificity of MAGL for ocular monoacylglycerols and the lack of desensitization in MAGL^−/−^ mice, suggest that the development of an optimized MAGL blocker offers therapeutic potential for treatment of elevated IOP.

Cannabinoids are best known as the psychoactive components of marijuana and hashish.^1^ However, these exogenous cannabinoids act on an endogenous signaling system that consists of receptors, ligands, and enzymes that produce and break down these ligands.^2^ This cannabinoid signaling system is distributed widely in the brain and elsewhere in the body.^3^ Though cannabinoids have a long history in the human pharmacopeia, one of the first modern uses for cannabinoids arose from the observation that the psychoactive component of marijuana (Δ^9^-THC) also reduces IOP.^4^ The prospect of cannabinoids as the basis of a therapy for glaucoma prompted a long series of studies that examined first the exogenous and then endogenous and synthetic cannabinoids (reviewed previously^5^).

Among the canonical cannabinoid receptors CB_1_ and CB_2_,^6,7^ abundant evidence has been presented for ocular CB_1_.^8–10^ Of the studies that have examined CB_2_ mRNA in the eye, only one has detected it.^9,11–13^ It is possible that the immune-related CB_2_ is upregulated under some pathologic conditions.^14^ Though there is evidence from a porcine culture model for a CB_2_ role in regulation of IOP, we have determined that CB_1_ receptors mediate most and perhaps the entire drop in murine IOP seen with synthetic cannabinoids.^15^ What is less well understood is the nature of the endogenous system in the anterior eye. Two arachidonic acid-based lipids, 2-arachidonoyl glycerol (2-AG) and arachidonoyl ethanolamide (AEA; aka anandamide^16,17^), are favored as the chief endocannabinoids, but the weight of evidence leans somewhat toward 2-AG as the chief endocannabinoid in neuronal signaling,^18^ though this may depend greatly on the brain and body region. Complicating the picture is evidence for anandamide as an endogenous ligand for transient receptor potential vanilloid 1 (TRPV1),^19^ while 2-AG also is able to activate the receptor, albeit more poorly.^20,21^ Both 2-AG and AEA have been found to reduce IOP^22–24^ as has the AEA congener palmitoylethanolamide^25^ (PEA) though the effect of PEA presumably does not occur via cannabinoid receptors, since, contrary to initial evidence for CB_2_,^26,27^ PEA does not activate CB_1_ or CB_2_.^28^

Beyond the identity of the endocannabinoids, there is the question of how they are produced and broken down. 2-Arachidonoyl glycerol is metabolized by any of several serine hydrolases (reviewed previously^29^). In brain homogenate, the bulk of hydrolysis occurs via monoacylglycerol lipase (MAGL) but α-β hydrolase domain 6 (ABHD6) and ABHD12 together accounted for approximately 20% of the hydrolysis of 2-AG in those samples.^30^ These experiments did not test fatty acid amide hydrolase (FAAH) or cyclooxygenase 2, each of which has been found separately to metabolize 2-AG.^31,32^ In principle, therefore, any combination of five different enzymes may be responsible for 2-AG metabolism^33^ and the question of which enzyme breaks down 2-AG in the anterior eye remains a matter of debate. Though MAGL accounts for the bulk of hydrolysis in brain lysates, the situation in the eye may prove very different. For instance, deleterious ABHD12 mutations have been noted in patients with polyneuropathy, hearing loss, ataxia, retinitis pigmentosa, and cataract (PHARC), a heritable disorder that includes retinitis pigmentosa and cataracts.^34^ This raises the possibility that ABHD12 may have a prominent role in the eye. Nor is this a purely academic question: targeting the enzymatic breakdown of endogenous cannabinoids (eCBs) has the potential to lower IOP, with consequent therapeutic potential. One study purportedly confirmed the role of MAGL in IOP in a porcine culture model using LY2183240, a compound described by the authors as a MAGL blocker.^35^ Instead, LY2183240 is an eCB uptake inhibitor as well as a nonspecific serine hydrolase inhibitor that is especially effective at blocking FAAH.^36^ Cannabinoid uptake inhibitors have been shown previously to lower IOP^37^ as has anandamide,^23,38^ which is an endogenous metabolic substrate of FAAH.^32^ Therefore, the question of a MAGL role remains unresolved. We tested two MAGL blockers, JZL184^39^ – the first selective blocker identified for this serine hydrolase, and KML29 – a potent MAGL blocker described more recently.^40,41^ 2-Arachidonoyl glycerol has been shown to lower IOP in a rabbit model, doing so biphasically, with an initial increase followed by a decline,^24^ but curiously, the effects of 2-AG were not blocked by a CB_1_ antagonist.

Using normotensive mouse models, including knockouts (KOs) for several relevant genes and their protein products, we now present evidence for a functional MAGL role in the regulation of endocannabinoid metabolism in the eye and IOP.

## Methods

### Animals

Experiments were conducted at the Indiana University campus. All mice used for IOP experiments were handled according to the guidelines of the Indiana University animal care committee, and in accordance with the ARVO Statement for the Use of Animals in Ophthalmic and Vision Research. Mice (age, 3–8 months) were kept on a 12-hour (6 AM–6 PM) light dark cycle, and fed ad libitum. Male C57BL/6J (C57) mice were obtained from Charles River Laboratories International, Inc. (Wilmington, MA, USA) or were kindly provided by Ken Mackie (Indiana University, Bloomington, IN, USA). Mice were allowed to acclimatize to the animal care facility for at least a week before their use in experiments. We used 64 animals in these experiments. CB_1_^−/−^, CB_2_^−/−^, and MAGL^−/−^ mice were kindly provided by Ken Mackie. All KOs are global KOs. CB_1_^−/−^ animals were received originally from Catherine Ledent (Catholic University, Leuven, Belgium) as heterozygotes.^42^ The CB_2_^−/−^ mice originally were purchased from Jackson Laboratories (Bar Harbor, ME, USA). The MAGL^−/−^ mice have been characterized previously in our laboratory^31^ and were developed in the laboratory of Ben Cravatt (Scripps Research Institute, La Jolla, CA, USA).

### Immunohistochemistry

Adult mice (CD1 strain or C57/BL6 strain, >5 weeks, of either sex, from breeding colony) were housed under a 12/12-hour day/night cycle then killed (during the light cycle) by rapid cervical dislocation. Eyes were removed, and the posterior eye section cut away, forming an eyecup, from which the lens was extracted. For immunocytochemistry, the anterior eyecup was fixed in 4% paraformaldehyde for an hour followed by a 30% sucrose immersion for 24 to 72 hours at 4°C. Tissue then was frozen in optimal cutting temperature (OCT) compound and sectioned (15–25 μm) using a Leica CM1850 cryostat. Tissue sections were mounted onto Fisher Superfrost-plus slides, washed, blocked with Sea Block blocking buffer (Thermo Fisher Scientific, Waltham, MA, USA) for 30 minutes, treated with a detergent (Triton X-100, 0.3% or saponin, 0.1%) and normal goat serum (0.5%), followed by primary antibodies overnight at 4°C. Secondary antibodies (Alexa488, Alexa 594 or Alexa647, 1:500; Invitrogen, Carlsbad, CA, USA) were subsequently applied at room temperature for 1.5 hours. CD1 sections were used to observe nonpigmented sections but were confirmed with C57 and KO controls. All primary antibodies, the immunogen used in their generation, their sources, and their working dilutions have been reported previously.^43^ The MAGL antibody was generously provided by Ken Mackie (Indiana University).

### IOP Measurements

Intraocular pressure was measured in mice by rebound tonometry, using a Tonolab (Icare Finland Oy, Helsinki, Finland). This instrument uses a light plastic-tipped probe to briefly make contact with the cornea; after the probe encounters the eye, the instrument measures the speed at which the probe rebounds to calculate IOP.

To obtain reproducible IOP measurements, mice were anesthetized with isoflurane (3% induction). The anesthetized mouse then was placed on a platform in a prone position, where anesthesia was maintained with 2% isoflurane. Baseline IOP measurements were taken in both eyes. A “measurement” consisted of the average value of six readings. One eye then was treated with drug (dissolved in Tocrisolve [Tocris Biosciences, Bristol, United Kingdom], a soya-based solvent,^44^ 5 μL final volume applied topically) while the other eye was treated with vehicle. The animal then was allowed to recover. After an hour, the animal was anesthetized again as above. Intraocular pressure then was measured in the drug-treated and vehicle-treated contralateral eye. In principle, anesthesia may alter IOP; however, our tests of IOP in contralateral eyes of the same animal means that observed effects are independent of hypothesized anesthetic effects on IOP. After a 2-week clearing period, animals were made available for measurement of IOP under other experimental conditions.

Intraocular pressure measurements following drug administration were analyzed by unpaired *t*-tests comparing drug-treated to vehicle-treated eyes. In one experiment, animals were injected with drug (JZL184, 4 mg/kg in saline, intraperitoneally). In this case, ocular pressures of animals were compared to those of vehicle-injected animals.

### Lipid Extraction

Mice were killed via cervical dislocation and both eyes were removed immediately and placed in an Eppendorf tube on dry ice. The eyes then were stored at −80°C. To begin the lipid extraction,^45,46^ samples were shock frozen in liquid nitrogen, which allowed them to be easily removed from the Eppendorf tube and weighed before being transferred to a 15 mL centrifuge tube. The mass of the largest sample was multiplied by 50 to determine how many milliliters of high performance liquid chromatography (HPLC)–grade methanol (Avantor Performance Materials, Inc., Center Valley, PA, USA) was to be added to the centrifuge tube. Then, 5 μL of vortexed 1 μM deuterium-labeled *N*-arachidonoyl glycine (d_8_NAGly; Cayman Chemical, Ann Arbor, MI, USA) was added to each test tube to serve as an internal standard. The spiked tubes were covered with Parafilm and were allowed to sit in the covered ice bucket for 2 hours. The eyes then were briefly homogenized using a sonicator (VirTis, Gardiner, NY, USA). Then, samples were spun in a centrifuge at 19,000*g* for 20 minutes at 20°C.

After centrifugation, supernatant was poured from the centrifuge tubes into 15 mL polypropylene tubes. Enough HPLC H_2_O (EMD Millipore Corporation, Billerica, MA, USA) to make a 75:25 water-to-organic solution was added to the supernatant. To partially purify the supernatant/water solution, solid phase extraction columns were used. One solid-phase 500 mg C18 extraction cartridge (Agilent Technologies, Lake Forest, CA, USA) for each tube of extract was inserted into a Preppy vacuum manifold apparatus located in a fume hood. To activate the hydrophobic carbon chains in the column, 5 mL of HPLC methanol was added to each column. When the methanol almost reached the bottom of the columns, 2.5 mL of HPLC H_2_O was added to the columns to activate the polar silica in the columns. When the water had almost run through the column, the supernatant/water solution was added and allowed to drip slowly through the column. After the solution had eluted, another 2.5 mL of HPLC H_2_O was added to the columns to wash off impurities. Then, 1.5 mL of 40% methanol was added to the column to wash off more impurities. The 40% methanol was allowed to elute completely and any eluate in the collector vials was discarded. The collector vials then were replaced with labeled autosampler vials (Perkin Elmer, Waltham, MA, USA) that corresponded to each sample. A series of 4 elutions with 1.5 mL of 60%, 75%, 85%, and 100% methanol as the eluting solvent was performed to partially purify the lipids being measured. More polar lipids, such as PGE_2_ or PGF_2α_, were present in the 60% and 75% elutions. N-arachidonylglycine (NAGly) was present in the 85% elution, whereas the most nonpolar lipids, such as 2-AG and AEA, were present in the 100% elution. Vials of eluents were stored in the −80°C freezer until they were ready for analysis.

### HPLC/Tandem Mass Spectrometry (HPLC/MS/MS)

Samples were analyzed using an Applied Biosystems API 3000 triple quadrupole mass spectrometer (Applied Biosystems Sciex, Framingham, MA, USA) with electrospray ionization. Levels of each compound were determined by running each sample using a multiple reactions monitoring (MRM) method tailored for each amide family of compounds. Samples were loaded with an autosampler (Shimadzu, Kyoto, Japan), which injected 20 μL from each vial into the chromatography system for each method run. To chromatograph the samples, an XDB-C18 (Agilent Technologies) reversed phase HPLC analytical column was used, which was kept at 40°C by a column oven (HP, Palo Alto, CA, USA). Two different types of mobile phase were used. Mobile phase A consisted of 20%/80% (vol/vol) methanol/water and 1 mM ammonium acetate (Sigma-Aldrich Corp., St. Louis, MO, USA). Mobile phase B instead contained 100% methanol with 1 mM ammonium acetate. Every method run began with 0% mobile phase B, reached a state of 100% mobile phase B flowing at 0.2 mL/minute, and gradually returned to 0% mobile phase B. Before running batches of samples, the ionization source was allowed to reach its operating temperature of 500°C and every vial warmed to room temperature and was vortexed for approximately 30 seconds.

Analyses of the HPLC/MS/MS peaks were performed using Analyst software. Chromatograms were generated by determining the retention time of analytes with a [M-1] or [M+1] parent peak and a fragmentation peak corresponding to the programmed values. The retention time then was compared to the retention time of a standard for the suspected compound. If the retention times matched, then the concentration of the compound was determined by calculating the area under the curve for the unknown and comparing it to the calibration curve obtained from the standards. Extraction efficiency was calculated with the d_8_-NAGly spiked recovery vial as a standard. Concentrations in moles per gram adjusted for percent recovery from the KO animals were compared to wild-type concentrations using a 1-way ANOVA. All statistical tests were done using SPSS Statistics 22 (IBM, Armonk, NY, USA). Statistical significance was defined as *P* ≤ 0.05 and a trending effect was defined as 0.05 < *P* ≤ 0.10. Unless otherwise noted, values are shown ± SEM.

### Drugs

We obtained 2-AG and JZL184 from Cayman Chemical (Ann Arbor, MI, USA), while KML29 and Tocrisolve were obtained from Tocris (Ellisville, MO, USA), and WIN55212-2 was obtained from Sigma-Aldrich Corp. Topically applied drugs were prepared by dilution in Tocrisolve.

## Results

### 2-AG Reduces IOP in a Concentration- and CB_1_-Dependent Manner

**Figure 1:**
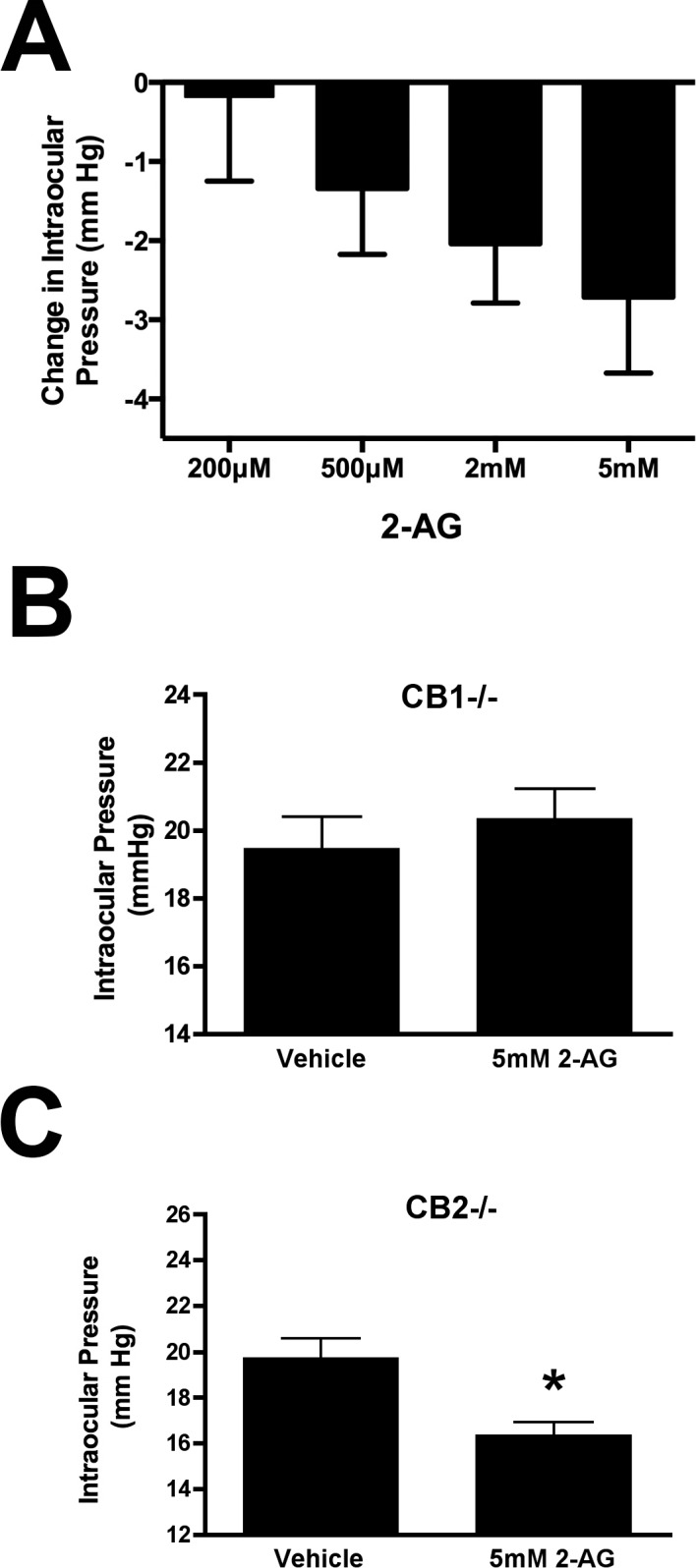
2-Arachidonoyl glycerol reduces IOP in a concentration- and CB1-dependent manner. (**A**) Shows changes in IOP (mm Hg) after 1 hour, with increasing concentrations of 2-AG lowering IOP in a concentration-dependent manner. (**B**) Responses to 5 mM 2-AG are absent in CB1^−/−^ animals, but present in CB2^−/−^ animals (**C**).

As noted above, 2-AG has been tested in a rabbit model and found to lower IOP, but the effect was not blocked by a CB_1_ antagonist.^24^ Using a mouse model, partly to take advantage of transgenic KO mice, we tested a range of concentrations of 2-AG (200 μM, 500 μM, 2 mM, and 5 mM, *n* = 8, 5, 8, 14), applied topically (Fig. 1A) and tested for their effect after 1 hour. One hour was chosen because 2-AG is broken down rapidly by several serine hydrolases.^30,47^ Although the maximal depression of IOP coincided with the highest concentration of applied drug, we found that even 500 μM reduced IOP. However, 200 μM did not produce a statistically significant drop in IOP and was thereafter considered a subthreshold concentration of 2-AG for topical treatments.

We next tested whether the effect of 2-AG was maintained in the absence of CB_1_ or CB_2_ receptors. We found that the effect of 2-AG was absent in CB_1_^−/−^ mice (Fig. 1B; baseline, 19.5 ± 0.9 mm Hg; 5 mM 2-AG, 20.4 ± 0.9 mm Hg; *n* = 6; *P* > 0.05 *t*-test) but present in CB_2_^−/−^ mice (Fig. 1C; baseline, 19.3 ± 0.9 mm Hg; 5 mM 2-AG, 16.7 ± 0.6 mm Hg; *n* = 5; *P* < 0.05 *t*-test). This is consistent with our findings for the synthetic cannabinoid agonist WIN55212,^15^ though in that study we observed a potentiation with WIN55212 in CB_1_^−/−^ animals suggesting that the inhibition normally observed masks a separate potentiating action via a non-CB_1_–dependent mechanism. We did not observe a statistically significant potentiation by 2-AG in CB_1_^−/−^ indicating that 2-AG does not similarly increase IOP.

### Topically Applied MAGL Blocker KML29 But Not JZL184 Lowers IOP

**Figure 2:**
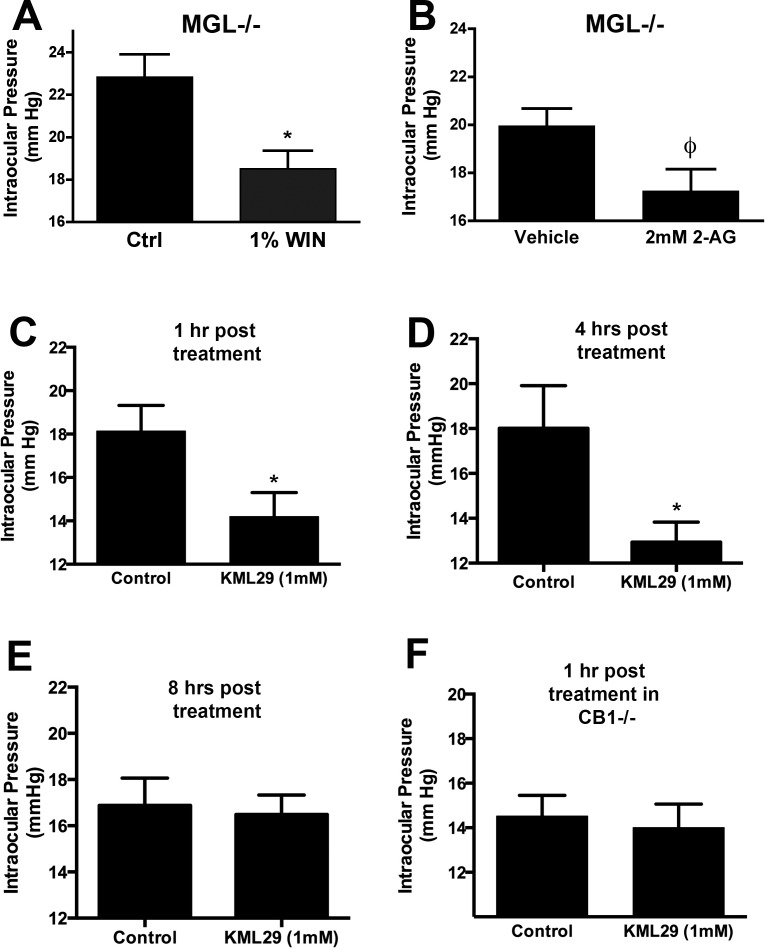
Monoacylglycerol lipase inhibition can reduce IOP. (**A**) Topical WIN55212 (1%) reduces IOP in MAGL^−/−^ eyes. (**B**) 2-Arachidonoyl glycerol (2 mM) treatment in MAGL^−/−^ eyes results in a trend toward IOP reduction (*P* = 0.0502). (**C**–**E**) Topical treatment with KML29 (1 mM) lowers IOP at 1, 4, and 8 hours after treatment. (**F**) However, KML29 is without effect in CB1^−/−^ mice. *P < 0.05, ^ϕ^*P* = 0.05, paired *t*-test.

As noted above, the bulk of 2-AG metabolism in the CNS occurs via MAGL.^30^ We first tested whether cannabinoid modulation of signaling was intact in MAGL^−/−^ mice. We have reported previously that MAGL deletion can result in desensitization of CB_1_, presumably due to the lack of 2-AG clearance from the vicinity of CB_1_ receptors.^47^ Baseline IOP in MAGL^−/−^ mice was comparable to WT (MAGL, 19.7 ± 1.5 mm Hg, *n* = 5; WT, 21.0 ± 0.9 mm Hg, *n* = 6). We found that the synthetic CB_1_ agonist WIN55212 (1%) continued to reduce IOP in MAGL^−/−^ mice (Fig. 2A; baseline, 22.8 ± 1.1 mm Hg; WIN55212, 18.5 ± 0.9, *n* = 6, *P* < 0.05 by paired *t*-test). However, 2 mM 2-AG did not lower IOP in MAGL^−/−^ eyes, though the effect trended toward statistical significance (Fig. 2B; baseline, 20.0 ± 0.7; 2 mM 2-AG, 17.3 ± 0.9, *n* = 11, *P* = 0.0502 by paired *t*-test).

Acute blockade of MAGL might potentiate endogenous 2-AG–mediated signaling. This was the case in autaptic hippocampal neurons.^33,47^ Therefore, we tested topical treatment with the selective MAGL blocker KML29 (1 mM) at 1 and 4 hours after treatment, finding that KML29 lowered IOP at 1 and 4 hours after treatment (Figs. 2C–D; control [1 hour], 18.1 ± 1.2; KML29 [1 mM, 1 hour], 14.2 ± 1.1; control [4 hours], 18.0 ± 1.9; KML29 [1 mM, 1 hour], 12.9 ± 0.9; *n* = 8, *P* < 0.05 by paired *t*-test). By 8 hours, we no longer observed an effect of KML029 (Fig. 2E; control [8 hours], 16.9 ± 1.2; KML29 [1 mM, 8 hours], 16.5 ± 0.8; *n* = NS by paired *t*-test). However, 300 μM KML29 reduced IOP at 1 hour but not at 4 hours (data not shown). The effect of 1 mM KML29 at 1 hour was absent in CB_1_^−/−^ mice, indicating that the effect of KML29 is CB_1_-dependent (Fig. 2F; control CB_1_^−/−^ [1 hour], 14.5 ± 0.9; KML29 CB_1_^−/−^ [1 mM, 1 hour], 14.0 ± 1.1; *n* = 5, not significant [NS] by paired *t*-test). The drop at 4 hours is substantial at 28%, in a normotensive model, indicating in an in vivo model that endogenous production of 2-AG can be harnessed for the purposes of lowering IOP. In separate experiments with MAGL blocker JZL184 we found that 1 mM of the MAGL blocker JZL184 did not lower IOP on its own, though it did lower IOP in combination when coapplied with a subthreshold concentration of 2-AG (data not shown) and when injected at 4 mg/kg (data not shown).

### Monoacylglycerols Are Elevated in Eyes of MAGL^−/−^ Mice

Lipidomics screens were performed in eyes of WT versus MAGL^−/−^ mice. Though most attention has focused on the canonical endcannabinoids, 2-AG and AEA, the body produces a range of related lipids. What function these might have still is largely an open question. A panel of approximately 80 lipids, a full list of which is found in Supplementary Table S1, was screened in wild type (WT) and KO mice. Three 2-acyl-glycerol MAGL substrates, including 2-AG, 2-oleoyl-sn-glycerol (2-OG) and 2-linolenoyl-sn-glycerol (2-LG), were detected in all samples. A total of 24 members of this panel included the *N*-oleoyl-, arachidonoyl-, palmitoyl-, stearoyl-, linoleoyl-, docosahexaenoyl-based *N*-acyl ethanolamines, *N*-acyl GABAs, *N*-acyl glycines, and *N*-acyl serines. The list additionally included linoleic and arachidonic free fatty acids, *N*-arachidonoyl taurine, and the prostaglandin metabolites PGE_2_ and PGE_2G_. As expected, by far the strongest increases were seen in the acyl-glycerols, all of which were substantially elevated. Most classes of lipids were unchanged, with a few exceptions that included anandamide, but the changes in levels of these lipids, though statistically significant, were modest. It has been shown previously that arachidonic acid metabolites, such as prostaglandins, are drastically altered when MAGL is blocked or eliminated.^48^ In mouse MAGL^−/−^ eyes, the prostaglandins were unaffected and arachidonic acid was not statistically significant though it did trend toward a decline. This is in striking contrast to steep declines in arachidonic acid and prostaglandin levels in spinal cord tissue from the same mice (see Table).

**Table:**
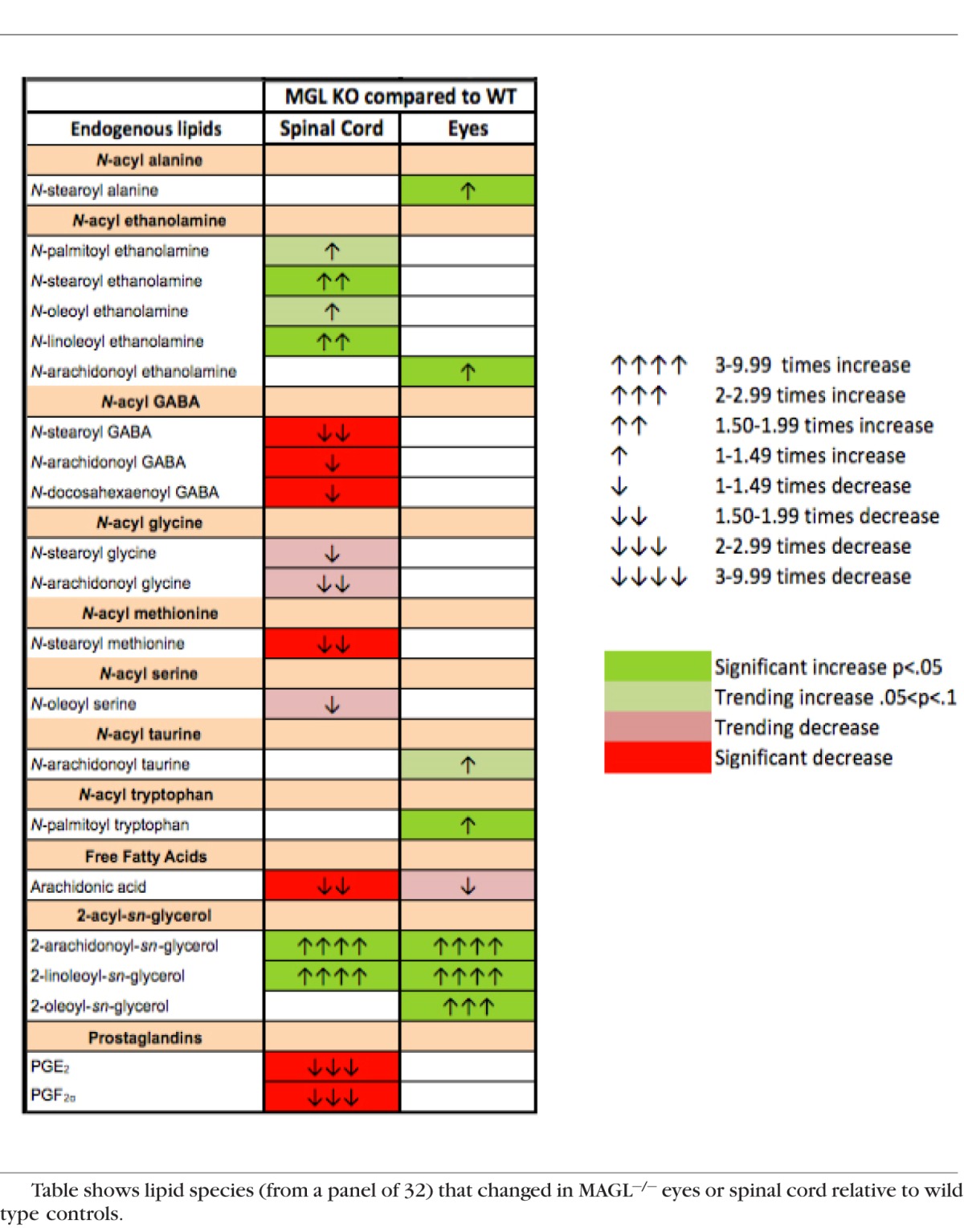
Monoacylglycerols Are Elevated in Eyes of MAGL^−/−^ Mice

### Monoacylglycerol Lipase (MGL) is Expressed Prominently in Ciliary Epithelium and Iris But Not Cornea

**Figure 3:**
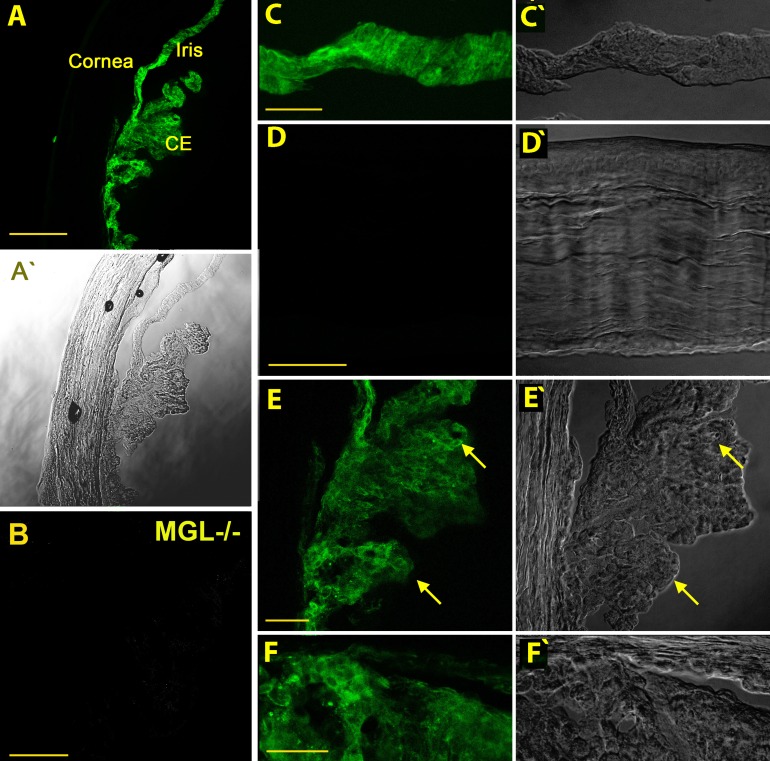
Monoacylglycerol lipase is prominently expressed in ciliary epithelium and iris but not cornea. (**A**) Overview in a nonpigmented CD1-strain mouse shows MAGL expression in several major anterior eye structures. CE, ciliary epithelium. (**A'**, **C'**–**F'**) Corresponding differential interference contrast (DIC) images. (**B**) Monoacylglycerol lipase staining in tissue from MAGL KO anterior eye tissue, taken at same settings. (**C**) Iris is prominently labelled. (**D**) Monoacylglycerol lipase is not present in corneal epithelium, endothelium, or stroma. (**E**) Monoacylglycerol lipase is present in ciliary epithelium (*arrow*), particularly the inner pigmented layer. (**F**) Close-up of the angle shows prominent labelling of the ciliary body (*lower portion* of image) but little labelling of the trabecular meshwork. *Scale bars*: (**A**) 150 μm, (**B**) 50 μm, (**C**–**E**) 40 μm, (**F**) 25 μm.

**Figure 4:**
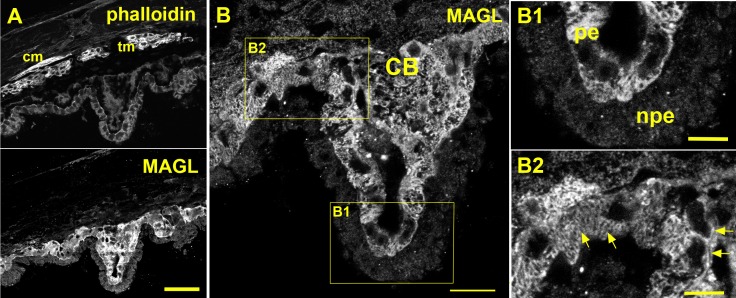
Monoacylglycerol lipase is expressed in pigmented ciliary epithelium. (**A**) *Top* shows phalloidin staining, particularly of ciliary muscle (cm) and trabecular meshwork (tm). *Bottom* shows MAGL staining absent outside of the ciliary body. (**B**) *Inset* from (**A**) shows MAGL staining in ciliary epithelium. (**B1**) *Inset* from (**B**) shows staining is limited to the inner pigmented epithelium (pe) and absent from nonpigmented epithelium (npe). (**B2**) *Second inset* shows that some staining appears to be membrane associated (*sideways arrows*) but other MAGL staining appears to be intracellular, in some cases with a striated pattern (*angled arrows*). *Scale bars*: (**A**) 50 μm, (**B**) 20 μm, (**C**, **D**) 5 μm.

We used immunohistochemical tools to examine the protein distribution of MAGL in the anterior chamber. Monolacylglycerol lipase is broadly but selectively distributed in the WT mouse anterior eye (e.g., ciliary epithelium, iris; Fig. 3A), staining that was absent in the MAGL KO mouse (Fig. 3B). Monolacylglycerol lipase was conspicuously absent from the cornea (Fig. 3D), despite the previously reported presence of CB_1_.^15^ It is prominently expressed in ciliary epithelium, but in the inner pigmented layer, not in the outer layer (Fig. 3E, arrow). This is shown in greater detail in Figure 4B. The MAGL staining in the ciliary epithelium appears to be membrane-associated but also intracellular (Fig. 4B2, arrows).

## Discussion

In 1971, Hepler and Frank^4^ first demonstrated that marijuana has a salutary effect on IOP. This set in motion a long series of studies across the subsequent 40 years, studies that continue today. Because the physiologic target of marijuana remained unknown, initial work focused on the chief psychoactive ingredient THC and related phytocannabinoids. With the identification of the cannabinoid CB_1_ and CB_2_ receptors and candidate endocannabinoids 2-AG and anandamide, these receptors and ligands became the target of most subsequent studies. The current study represents a continuation of these inquiries, now examining eCBs in greater detail, with an emphasis on their degradation by the endogenous serine hydrolases most implicated in the breakdown of 2-AG, MAGL.^39,47^ Our major findings are that 2-AG reliably lowers IOP in a concentration- and CB_1_-dependent manner and that MAGL blockade can be harnessed to lower IOP.

Thanks to recent advances in our understanding of the synthesis, metabolism, and regulation of endocannabinoids we now know much more about the enzymes that produce or break down eCBs, particularly in the case of 2-AG (reviewed by Murataeva et al.^29^). MAGL remains the enzyme most strongly-implicated in 2-AG metabolism, particularly in the central nervous system (CNS),^30,48^ but several other enzymes (ABHD6, ABHD12, COX2, or FAAH) may have a role (perhaps a dominant role) depending on tissue and subcellular distribution of these proteins. Therefore, it has remained an open question which of these has a primary role in degrading ocular 2-AG. Indeed, it may prove to be the case that the roles are shared by multiple enzymes, or that they vary by tissue even within the eye. That might, in fact, be a desirable outcome, since each of these proteins represents a potential distinct therapeutic target. Because CB_1_ is expressed in many ocular tissues,^49^ where it presumably has distinct roles, one risk of therapeutic intervention with a simple CB_1_ agonist is the likelihood of off-target activation and attendant side-effects. Our study has explored the possibility of raising endogenous eCB levels by blocking their degradation by MAGL to lower IOP.

As noted above, our data strongly support 2-AG as the eCB acting via CB_1_ to reduce IOP. Arachidonoyl ethanolamide was identified 3 years before 2-AG,^17^ but it has some unusual properties. It generally is found at far lower concentrations than 2-AG in nervous tissue^32^ and is a full agonist at TRPV1 receptors, members of an unrelated class of ion channels.^50^ Monoacylglycerol lipase is closely linked with 2-AG, often colocalized with CB_1_ including in retina.^43^ Monoacylglycerol lipase determines the time course of depolarization-induced suppression of excitation/inhibition (DSE/DSI),^33,47^ while 2-AG levels rise dramatically in the CNS of MAGL^−/−^ animals.^51^

One encouraging finding, in terms of therapeutic relevance, is that CB_1_ IOP effects do not desensitize in MAGL^−/−^ mice. Desensitization of responses is a risk with any therapy and MAGL deletion had been found to result in diminished CB_1_ binding in some, but not all, brain regions of mice^51^ as well as desensitization in neurons.^47^

Also notable is the finding that MAGL enzyme activity appears to be relatively restricted to the monoacylglycerols; only these were dramatically increased in eyes of MAGL^−/−^ mice. This is in contrast to the striking perturbation of arachidonic acid and prostaglandin levels that we see in the spinal cords of the same mice. As noted above, it has been reported previously that MAGL blockade or elimination substantially reduces arachidonic acid and prostaglandins.^48^ 2-Arachidonoyl glycerol is metabolized by MAGL into arachidonic acid and glycerol and it appears that in much of the CNS this 2-AG–derived arachidonic acid serves as a substrate for prostaglandin synthesis and that MAGL inhibition risks “locking up” arachidonic acid, making it unavailable for production of prostaglandins. This does not appear to be a major factor in the eye. The strong increases in not only 2-AG but also the related lipids 2-LG and 2-OG does raise the question of what role these lipid species might have in the eye. Compounds, such as 2-LG and 2-OG, have been proposed to act as entourage compounds,^52^ but 2-OG instead may be an agonist at GPR119.^53^

Monoacylglycerol lipase protein was shown here to be selectively distributed in the eye, chiefly in pigmented ciliary epithelium and iris. Given its role in the metabolism of a larger number of acyl glycerol lipids, MAGL may have multiple roles in the eye, including those that are unrelated to CB_1_ signaling. We have shown previously that CB_1_ is widely distributed in several regions that are implicated in regulation of IOP, including ciliary epithelium and trabecular meshwork.^49^ Of the tissues most prominently expressing MAGL, the one most likely to have a role in regulating IOP is the ciliary epithelium. It is possible that MAGL blockade elevates 2-AG more broadly in the anterior chamber and that the site of action of this elevated 2-AG is elsewhere in the anterior eye.

Our findings that 2-AG inhibits IOP in a CB_1_-dependent manner are inconsistent with the findings of Laine et al.^24^ The CB_1_ antagonist used by Laine et al.,^24^ AM251, did block effects by other agonist in the same study and, therefore, was presumably effective. Therefore, the difference in findings may be due to species differences, an issue that will need to be explored in future studies. Our results contradict the findings of Njie et al.,^35^ who reported a role for CB_2_ in regulating aqueous humor outflow. There is some evidence for CB_2_ in the anterior eye consisting of a series of studies from one research group using a porcine culture model for which KO controls are unavailable,^35,54,55^ but this is opposed by studies from several other groups that have sought but not detected CB_2_ receptors or function.^12,13,15^ Though the use of CB_2_ KOs by Hudson et al.^15^ and here are a strong argument against a measurable CB_2_ role in regulation of IOP, it is possible that under certain conditions, such as in a culture model or due to species differences, CB_2_ is upregulated and, therefore, has a role under limited conditions. It should be noted however, that some of the evidence for a CB_2_ role (e.g., see the report of Zhong et al.^55^) relies on the ostensible CB_2_-selectivity of JWH015, a compound that we have determined to be an efficacious CB_1_ agonist.^56^ Similarly, as noted in the introduction, the same group made use of LY2183240 claiming that it was a MAGL blocker,^35^ whereas it had been described previously as a nonspecific blocker of serine hydrolases with activity at FAAH, in addition to being an eCB uptake inhibitor.^36^ The activity at FAAH may account for their observed effects. In contrast, the current study has made use of the extensively characterized JZL184,^39^ used in >100 published studies, and the more recently described KML29^40,41^ in addition to MAGL KO animals to resolve the question of a MAGL role.

The eye offers distinct advantages for therapeutic intervention. Because of its relative isolation from the rest of the body, an agent that can be applied topically offers an opportunity to intervene selectively in the eye. This reduces but does not eliminate the risk of deleterious side effects for a widely distributed receptor, such as CB_1_, and eye drops come with their own risks. However, the use of a MAGL blocker is attractive and under active investigation for several physiologic conditions, such as pain.^57^ As such, lowered IOP may be a beneficial side effect of any systemic MAGL blocker-based treatment; effects on IOP certainly will need to be taken into consideration.

Although several drug classes are available that lower IOP,^58,59^ not all patients respond to these drugs, and those who do are not always responsive during the full course of their treatment. Importantly, a study of patients with unresponsive forms of glaucoma found that these patients responded well to cannabinoid-based therapy using a synthetic CB_1_ agonist.^60^ In an exploration of the potential to harness eCBs for the purpose of lowering IOP we offer several insights. Blockade of the chief 2-AG metabolizing enzyme MAGL lowers IOP, doing so without evidence of CB_1_ desensitization even in the case of MAGL deletion. Monoacylglycerol lipase appears to be relatively specific for monoacyglycerols in the eye and does not perturb global arachidonic acid levels or of the prostaglandins tested. Lastly, our data lend further support for 2-AG as the eCB mediating cannabinoid reduction of IOP: blockade of the chief 2-AG metabolizing enzyme lowers IOP. Taken together, our results expand our understanding of eCB metabolism and raise the possibility of a therapeutic approach to IOP that takes advantage of the naturally occurring eCB.

## Supplementary Material

Supplement 1
